# Using Contemplative Medicine to Harness Compassion in the Palliative Care Setting: Lessons Learned

**DOI:** 10.1089/pmr.2024.0020

**Published:** 2024-12-04

**Authors:** Milagros D. Silva, Brigit C. Palathra

**Affiliations:** ^1^ Division Geriatrics and Palliative Medicine, Weill Cornell Medicine, New York, New York, USA.; ^2^ Department of Palliative Care, Mount Sinai South Nassau, Oceanside, New York, USA.

**Keywords:** burnout, contemplative medicine, mindfulness, physician wellness

## Abstract

**Background:**

Burnout is common among palliative care clinicians caring for patients with a serious illness. Contemplative medicine is an emerging approach that aims to utilize Buddhist concepts of mindfulness, insight, and compassion to address unspoken suffering in clinicians.

**Objectives:**

To introduce and share contemplative medicine practices with Hospice Palliative Medicine (HPM) fellows participating in two academic programs in New York.

**Methods:**

Pilot educational sessions in contemplative medicine were conducted following a Contemplative Medicine Fellowship’s relationship-centered and cohort-based curriculum. A short survey assessing HPM fellows’ attitudes toward core competencies in contemplative medicine was administered to seven HPM fellows.

**Results:**

Participants agreed that being present with those who are suffering are healing acts by themselves and that contemplative medicine can complement HPM fellows’ skillsets when providing care to patients with serious illnesses. Common themes like “being awake” and “low self-compassion” were discussed by participants during the sessions. Techniques like pausing and mindful breathing were found helpful to practice throughout a busy workday.

**Discussion:**

Incorporating contemplative medicine practices into an HPM fellowship may provide opportunities to (1) promote learner emotional development and (2) teach learners self-awareness of how difficult emotions can affect communication with patients.

## Introduction

Compassion in medicine is considered a cornerstone of quality health care.^1,2^ However, many clinicians are unable to interact compassionately with patients and families because of competing institutional priorities, an overfocus on quantitative health care metrics, and the very structure of the increasingly hurried health care system.^2,3^ For example, compassionate encounters can be time consuming, unpredictable to schedule, and resource intensive, and therefore be viewed as financially nonviable. When health care professionals are unable to achieve the goal of compassionate care, they may experience a perceived loss of control, diminished morale, and decreased ability to cope with their work environment.^3,4,5^ This can lead to burnout, potentially further perpetuating a cycle that deemphasizes compassionate care.

Burnout is particularly common among palliative clinicians caring for patients with serious illnesses,^6,7^ and the inability to provide compassionate care may contribute to this phenomenon. A survey of fellowship-trained palliative physicians found the burnout rate to be high (52%).^8^ Palliative care clinicians manage symptoms caused by a life-limiting or serious illness in various care settings and regularly witness the suffering of patients and their loved ones.^6,7,9^ Many strategies have been employed to relieve the distress resulting from this work, including emotional regulation techniques, as the very nature of the work can result in the internalization of sadness, anxiety, or fear.^10,11^

Research has shown that mindfulness can help decrease burnout and assist in the management and reduction of stress in patients generally.^7,12,13^ Therapeutic interventions involving mindfulness have risen dramatically in the past decade. For example, a PubMed search of therapeutic application of “mindfulness” yielded over 3800 results in the year 2022 compared to 70 in 2008.^3^ However, we are unaware of mindfulness techniques specifically applied to this problem.

Contemplative medicine aims to utilize Buddhist concepts of mindfulness, insight, and compassion to address unspoken suffering in clinicians. Fundamental precepts of Buddhism include the fostering compassion of the self, of others, and the community in general.^12,13^ In other contexts, mindfulness and meditation have been shown to help health care professionals cope with stress, improve self-care, and improve patient care.^7,12,13,14,15^

The objective of this educational pilot was to introduce contemplative medicine concepts and practice to Hospice Palliative Medicine (HPM) trainees in an academic fellowship program.

## Methods

### Participants and educational sessions

We designed pilot educational sessions in contemplative medicine (Supplementary Appendix SA1) aimed at HPM fellows to introduce basic principles and discuss how to adapt contemplative medicine in the clinical setting. The five sessions followed the Contemplative Medicine Fellowship’s curriculum developed by the New York Zen Center for Contemplative Care.^16^ The Contemplative Medicine Fellowship aimed to cultivate resiliency by integrating a contemplative approach to the practice of medicine. The methodology utilized was a cohort-based and relationship-centered learning model.^15^ Institutional review board (IRB) approval was not obtained as it was determined not human subjects research by the Weill Cornell Medicine IRB.

The sessions were delivered over the period April 2022 to June 2022. The joint sessions were conducted at two separate campuses within a major health care system in New York City. Two attending physicians (B.C.P. and M.D.S.), who were completing a 12-month training in Contemplative Medicine Fellowship, served as facilitators for the educational sessions. One facilitator (M.D.S.) was faculty at one of the training programs and the other facilitator (B.C.P.) was an HPM fellowship program director at the other site. Current HPM fellows from each of their respective campuses were invited to join for 45–60 minutes on a weekly basis for five sessions.

All of the sessions were voluntary and not part of the established academic fellowship curriculum. Mindful breathing was incorporated at the beginning and end of each session. Eventually, HPM fellows were asked to lead the sessions with mindful breathing. In each session, a reflective dialogue model was utilized to allow for conversation, thought-sharing, and active listening between the participants.^17,18^ The two facilitators presented important “pearls” and questions that allowed for reflection on the personal, interpersonal, and clinical experiences of the intended learners. Each session was conducted as a shared joint session between the campuses using Zoom. This format allowed for community building, cross-pollination of thoughts, and encouragement of shared experiences.

Throughout the sessions, open-ended questions were utilized as conversation prompts. The group was asked questions such as “Are you awake?,” “What aspects of our lives cause us to suffer?,” “How are we pausing?,” “What are you feeling in this moment?,” “How do we have compassion for ourselves?,” and “How do we bring curiosity to a patient encounter?”

### Survey development

A short pre-curriculum survey (Supplementary Appendix SA2) was administered to estimate the frequency with which the HPM fellows endorsed specific attitudes about core competencies in contemplative medicine. One item employed Likert scale responses. A second part of the survey employed a qualitative open-ended question to define contemplative medicine. We also ascertained HPM fellows’ demographic status including age, gender, and race/ethnicity. All survey items were developed *de novo*. The survey was administered anonymously, using Qualtrics XM software (Qualtrics, Provo, UT). We did not compare responses between campuses given the small sample size.

### Statistical analysis

Descriptive statistics were performed for all numeric and categorical variables. For open-ended survey responses, inductive content analysis was used.^19^ The two facilitators who led the training recorded field notes of comments made by participants during each teaching session. We analyzed the qualitative data using content analysis. A codebook was developed inductively and revised throughout the data analysis process. During open coding, two co-authors (M.D.S. and B.C.P.) independently coded the qualitative data, identifying sections of text representing discrete concepts and applying code. A final set of codes was compared, developed, and organized into discrete themes.

## Results

### Participant characteristics

An invitation was extended to seven HPM fellows in training during the 2021–2022 academic year. A total of five participants completed the survey at the start of the educational sessions (Table 1).

**Table 1.** Demographic Characteristics of Participants

	Total (*n* = 5) *N* (%)
Age (years)	
21–34	4 (80%)
35–44	1 (20%)
45 and above	0
Gender	
Man	1 (20%)
Woman	4 (80%)
Prefer to self-describe	0
Prefer not to say	0
Race/ethnicity	
Asian or Asian American	3 (60%)
White or Caucasian	2 (40%)
American Indian or Alaska Native	0
Black or African American	0
Hispanic or Latinx	0
Multiracial or biracial	0
Native Hawaiian or Pacific Islander	0
Other	

### Themes that emerged during sessions

Four themes emerged from the analysis of the qualitative data. The first theme was “being asleep throughout medical training,” which was described by a participant as “going through the motions” without paying attention to their lived experiences. Participants shared emotions of feeling rushed and distracted during medical training and at work. Such themes led to further reflection on emotions such as regret, loss, grief, and hope. Both facilitators and HPM fellows shared their experiences with “being awake.” Each participant shared how they are trying to remain awake to live to the fullest, appreciate family and friends, and enjoy other important life events.

The second theme of “pausing throughout the day” was raised as a tool to help stay grounded, focused, re-charged, and connected. Furthermore, reflection occurred on how pausing could have helped in certain past scenarios and the effect it had on other personal experiences.

The third theme was “mindful breathing for self-care,” which was viewed by HPM fellows as a tool to stay grounded and manage stress. An HPM fellow reported the action made them feel calm and led to a positive feeling. One HPM fellow shared “you need to breathe to live” and described the simple but basic need to breathe as an important tool to be aware of throughout their busy workdays.

The last theme was “low self-compassion.” One participant shared the innate tendency of physicians to be high achievers, which often leads to “being too hard on oneself.” A discussion was held to reflect on ways we are being kind or can be kind to ourselves. One HPM fellow suggested the notion of being more aware that one is trying to do their best and “accepting their best as enough.” Other participants shared their opinions about empathy and compassion. One of the sessions was spent discussing differences in empathy and compassion in their personal versus professional lives.

### Survey answers

Participants were asked to define contemplative medicine in their own words. One participant defined it as “the provision of medical care to patients in a way that is deliberate and considerate, taking into account all aspects of their illness.” The majority of participants described contemplative medicine as a medical practice characterized by “mindfulness,” “being thoughtful,” “self-reflecting,” and “being introspective.” One participant shared that a value of contemplative medicine is that it “complements our skillset of providing care to patients” and can include “prayer, yoga, etc.”

All participants “somewhat agreed” to “strongly agreed” that they acknowledged their own limitations and felt confident in the skill of listening deeply to patients and family. Similarly, all participants “somewhat agreed” to “strongly agreed” that being present with those who are suffering are healing acts by themselves. When asked to rate the statement “it’s not enough if I’m not able to treat or fix a patient’s physical distress,” 40% (*n* = 2) participants “somewhat agreed” to “strongly agreed” There was variability in answers when asked how difficult emotions affected their ability to provide appropriate care or communicate effectively (Table 2).

**Table 2.** Attitude Questions

	Total (*n* = 5) *N* (%)		
	“Strongly disagree” to “somewhat disagree”	“Neither”	“Strongly agree” to “somewhat agree”
Being present with those who are suffering are healing acts in themselves	0 (0%)	0 (0%)	5 (100%)
It’s not enough if I’m not able to treat or fix a patient’s physical distress (pain, nausea, etc.)	3 (60%)	0 (0%)	2 (40%)
I am confident in the skill of listening deeply to patients and families	0 (0%)	0 (0%)	5 (100%)
Difficult emotions (frustration, sadness, guilt, etc.) during patient encounters interfere with my ability to provide appropriate care	2 (40%)	1 (20%)	2 (40%)
Difficult emotions (frustration, sadness, guilt, etc.) during patient encounters interfere with my ability to communicate effectively	1 (20%)	2 (40%)	2 (40%)
I acknowledge my own limitations	0 (0%)	0 (0%)	5 (100%)

## Discussion

The pilot educational sessions aimed to introduce contemplative medicine concepts and practices to HPM trainees in an academic fellowship program. The current HPM training paradigm emphasizes patient and family care, medical knowledge, practice-based learning and improvement, interpersonal and communication skills, professionalism, and systems-based practice.^20^ We believe that incorporating contemplative medicine practices into an HPM fellowship may provide opportunities to address all these domains by (1) promoting learner emotional development to improve clinical decision making, (2) teaching learners self-awareness of how difficult emotions affect communication and the provision of care for patients, and (3) encouraging learners to develop a layered community and to mobilize these relationships to address self-care, patient care, and system level practice.

Despite the new and emerging term “contemplative medicine,” all participants had their own definitions, which contained key descriptors such as “mindfulness” and “self-reflection.” All the participants also shared positive attitudes toward the concept of “being present in suffering as a healing act” and the skill of active listening and “bearing witness.” We speculate that the work of the fellows in HPM informs how they answered those questions.

All the participants shared they valued the sense of community being offered by the sessions, the opportunity to connect and have a safe space to explore their emotions and experiences. Tools such as pausing and mindful breathing were thought to have the potential of being helpful in their professional lives and clinical encounters. Kindness, love, and compassion toward oneself were concepts that participants found important to help them live healthy personal and professional lives.

Forty percent (*n* = 2) “somewhat agreed” to “strongly agreed” that not being able to fix a patient’s physical symptom is “not enough.” Fellows are frequently evaluated on medical knowledge and medical management decisions throughout residency, fellowship, and then during the certification process. Respondent answers may be based on the perceived importance of understanding pharmacologic, surgical, and interventional approaches to complex suffering and distress.^20,21,22^

Responses were divided regarding how difficult emotions affect communicating with and providing care to patients. Participants’ responses may reflect the current medical education and training, which focuses on the cognitive aspects of reasoning and decision making.^23^ A systematic review found an association between clinicians’ emotions and, consciously or unconsciously, affecting their clinical decision making.^23^ Undergraduate and graduate medical education do not routinely incorporate the assessment and development of emotional competence and emotional self-regulation though emotions are linked to clinical decision making. Ultimately, some learners may not be aware of how difficult emotions affect the provision of care. Additionally, how the participants self-regulate their emotions during clinical encounters is not known.

At the conclusion of the sessions, two HPM fellows participating at one campus requested that sessions continue weekly beyond the intervention endpoint. Both campuses are exploring opportunities to incorporate contemplative medicine into the curriculum of their HPM fellowship.

The authors acknowledge that this pilot is limited by its small sample size and the educational sessions were limited to five in total. The pilot was unique in that facilitators were current participants in a Contemplative Medicine Fellowship during the delivery of the five educational sessions; this may also limit generalizability. To replicate our study, new facilitators would need training in contemplative medicine concepts and experience in the reflective dialogue format. Formal measures specific to contemplative medicine evaluation would also need to be created. Future research should involve offering the curriculum to a larger group of HPM fellows and conducting formal pre- and post-intervention assessments to assess for satisfaction with the curriculum and impact on salient outcomes, including the level of mindfulness, coping, resilience, and professional quality of life. If found to be effective, further studies would be needed to determine the duration, frequency, and scalability of contemplative sessions during an HPM fellowship training year.

## Conclusion

In summary, the pilot educational sessions introducing contemplative medicine concepts and practices to HPM trainees highlight that further educational interventions in contemplative medicine should be developed and evaluated in academic training programs. The concept of contemplative medicine aims to utilize mindfulness, reflection, and compassion to address unspoken suffering in clinicians, which can as a result help health care professionals cope with stress, improve self-care, and prevent or reduce burnout.^15^

## Supplementary Material

Supplementary Appendix SA1

Supplementary Appendix SA2

## Abbreviations Used

HPM: Hospice Palliative Medicine
IRB: Institutional Review Board

## References

[1] de Zulueta P. How do we sustain compassionate healthcare? Compassionate leadership in the time of the COVID-19 pandemic. Clinics in Integrated Care 2021;8:100071; doi: 10.1016/j.intcar.2021.100071

[2] George CE. What might a good compassionate force protocol look like? AMA J Ethics 2021;23(4):E326–E334; doi: 10.1001/amajethics.2021.326 33950828

[3] Glisch C, Yadav S, Bhandari S, Jha P. Perceptions of burnout among academic hospitalists. WMJ 2021;120(4):268–272. 35025173

[4] Bourne DW, Epstein E. The experience of moral distress in an academic family medicine clinic. HEC Forum 2023;35(1):37–54; doi: 10.1007/s10730-021-09453-9 34050444

[5] West CP, Dyrbye LN, Shanafelt TD. Physician burnout: Contributors, consequences, and solutions. J Intern Med 2018;283(6):516–529; doi: 10.1111/joim.12752 29505159

[6] Kamal AH, Bull JH, Wolf SP, et al. Prevalence and predictors of burnout among hospice and palliative care clinicians in the U.S. J Pain Symptom Manage 2020;59(5):e6–e13; doi: 10.1016/j.jpainsymman.2019.11.017 31778784

[7] Horn DJ, Johnston CB. Burnout and self care for palliative care practitioners. Med Clin North Am 2020;104(3):561–572; doi: 10.1016/j.mcna.2019.12.007Reddy 32312415

[8] Reddy SK, Yennurajalingam S, Tanco K, et al. Frequency and prediction of burnout among physicians who completed palliative care fellowship training—A 10 year survey. J Pain Symptom Manage 2022;64(1):e15–e21; doi: 10.1016/j.jpainsymman.2022.02.009 35183705

[9] Sullender RT, Selenich SA. Financial Considerations of Hospital-Based Palliative Care. RTI Press: Research Triangle Park (NC); 2016. https://www.ncbi.nlm.nih.gov/books/NBK532652/ doi: 10.3768/rtipress.2016.rr.0027.1603 30398817

[10] Cho E, Cho HH. Factors influencing compassion fatigue among hospice and palliative care unit nurses. J Hosp Palliat Care 2021;24(1):13–25. doi: 10.14475/jhpc.2021.24.1.13 37675055 PMC10179998

[11] Portoghese I, Galletta M, Larkin P, et al. Compassion fatigue, watching patients suffering and emotional display rules among hospice professionals: A daily diary study. BMC Palliat Care 2020;19(1):23; doi: 10.1186/s12904-020-0531-5 32098618 PMC7043034

[12] Ludwig DS, Kabat-Zinn J. Mindfulness in medicine. JAMA 2008;300(11):1350–1352; doi: 10.1001/jama.300.11.1350 18799450

[13] Kalra S, Priya G, Grewal E, et al. Lessons for the health-care practitioner from Buddhism. Indian J Endocrinol Metab 2018;22(6):812–817; doi: 10.4103/ijem.IJEM_286_17 30766824 PMC6330872

[14] Cocker F, Joss N. Compassion fatigue among healthcare, emergency and community service workers: A systematic review. Int J Environ Res Public Health 2016;13(6):618; doi: 10.3390/ijerph13060618 27338436 PMC4924075

[15] Kennard A, Low Dog T, Clinkenbeard J, et al. Contemplative medicine: A practical approach to ‘Well-Being 2.0’ in medicine. Explore 2024;20(3):439–442; doi: 10.1016/j.explore.2023.11.002 37977922

[16] New York Zen Center. Contemplative Medicine Fellowship. Available from: zencare.org/contemplative-medicine-fellowship/

[17] Ng SL, Kinsella EA, Friesen F, et al. Reclaiming a theoretical orientation to reflection in medical education research: A critical narrative review. Med Educ 2015;49(5):461–475; doi: 10.1111/medu.12680 25924122

[18] Chu SY, Lin CW, Lin MJ, et al. Psychosocial issues discovered through reflective group dialogue between medical students. BMC Med Educ 2018;18(1):12; doi: 10.1186/s12909-017-1114-x 29321068 PMC5763939

[19] Hsieh HF, Shannon SE. Three approaches to qualitative content analysis. Qual Health Res 2005;15(9):1277–1288; doi: 10.1177/1049732305276687 16204405

[20] Barnett MD, Buckholz G, Christensen A, et al. Development of subspecialty-specific reporting milestones for hospice and palliative medicine fellowship training in the U.S. J Pain Symptom Manage 2020;60(1):151–157; doi: 10.1016/j.jpainsymman.2020.01.008 31988020

[21] von Gunten CF, Sloan PA, Portenoy RK, et al. Trustees of the American Board of Hospice and Palliative Medicine. Physician board certification in hospice and palliative medicine. J Palliat Med 2000;3(4):441–447; doi: 10.1089/jpm.2000.3.4.441 15859696

[22] Teoli D, Kalish VB. Palliative Care. In: StatPearls. StatPearls Publishing: Treasure Island (FL); May 4, 2022. 30725798

[23] Kozlowski D, Hutchinson M, Hurley J, et al. The role of emotion in clinical decision making: An integrative literature review. BMC Med Educ 2017;17(1):255; doi: 10.1186/s12909-017-1089-7 29246213 PMC5732402

