# Supplementary material for: Using Contemplative Medicine to Harness Compassion in the Palliative Care Setting: Lessons Learned
Source: Palliat Med Rep. 2024 Dec 4;5(1):537–42. doi: 10.1089/pmr.2024.0020 (PMC11693954; doi:10.1089/pmr.2024.0020)
Supplement: Supplementary Appendix SA1 [file pmr.2024.0020_suppl_datas1.docx]

Appendix B. Attitudes Survey

1. Demographics:

Age

- 21 to 34
- 35 to 44
- 45 to 54
- 55 and over

Gender

- woman
- man
- prefer to self describe: _______
- prefer not to answer

Which of the following best describes your race/ethnicity?

- Asian or Pacific Islander
- Black or African American
- Hispanic or Latinx
- Native American or Alaskan Native
- White or Caucasian
- Multiracial or Biracial
- Race/ethnicity is not listed here

1. Attitude questions
2. How would you describe contemplative medicine?

Please indicate how much you agree with the following statements:

|  | Strongly disagree | Disagree | Neither disagree nor agree | Agree | Strongly agree |
| --- | --- | --- | --- | --- | --- |
| Being present with those who are suffering are healing acts in themselves |  |  |  |  |  |
| It’s not enough if I’m not able to treat or fix a patient’s physical distress (pain, nausea, etc) |  |  |  |  |  |
| I am confident in the skill of listening deeply to patients and families |  |  |  |  |  |
| Difficult emotions (frustration, sadness, guilt, etc) during patient encounters interfere with my ability to provide appropriate care |  |  |  |  |  |
| Difficult emotions (frustration, sadness, guilt, etc) during patient encounters interfere with my ability to communicate effectively |  |  |  |  |  |
| I acknowledge my own limitations |  |  |  |  |  |
