# Supplementary material for: Using Contemplative Medicine to Harness Compassion in the Palliative Care Setting: Lessons Learned
Source: Palliat Med Rep. 2024 Dec 4;5(1):537–42. doi: 10.1089/pmr.2024.0020 (PMC11693954; doi:10.1089/pmr.2024.0020)
Supplement: Supplementary Appendix SA2 [file pmr.2024.0020_suppl_datas2.docx]

Appendix A. Educational Session

Session #1

● Mindful Breaths x3

● Checking in with each other.

○ How are you feeling at this moment?

○ Are you “awake’?

○ Are you distracted? Why?

● Introduction to Contemplative Medicine

○ What do you understand about Contemplative Medicine?

○ Explore the First and Second Noble Truths – discussion that suffering exists and exploring the cause of suffering in our personal and clinical lives.

○ What aspects of our lives cause us to suffer?

■ What triggers you?

■ What pushes your buttons?

■ Where do you think that comes from?

○ What emotions arise in you when you are faced with difficult patient encounters (dying, anger, pain, loss)

○ Think about a clinical encounter when we were faced with suffering. Were you trying to fix it or find a solution?; Were you being present with the suffering? accepting things just as they are?

● Mindful Breaths x3

Session #2

● Mindful Breaths x3

● Checking in with each other.

○ How are you feeling at this moment?

○ Are you “awake’?

○ Are you distracted? Why?

● Explore the importance of pausing throughout the day.

○ Using the pause to keep balance and your “tank full”

○ Thinking about the last 1-2 weeks, can you share a specific example where a pause could have helped?

○ What hinders you from pausing? Why?

○ How do you think it can help you with your professional and personal roles? ○ How did you pause and what did it feel like?

● Mindful Breaths x3

Session # 3

● Mindful Breaths x3

● Checking in with each other.

○ How are you feeling at this moment?

○ Are you “awake’?

○ Are you distracted? Why?

● Explore the concept of *BEGINNER'S MIND or NOT KNOWING (bring curiosity to the encounter)*

○ How can ‘not knowing’ bring freshness to an encounter?

○ Reflect on a patient encounter or colleague interaction where you felt challenged?
■ What triggered you in the interactions?

■ What pushed your buttons?

■ Were you actively listening and open to the ideas of this person?

■ How did you feel at the end of the interaction?

○ Can you think of an example when you brought a receptive mind to this interaction?

■ Did curiosity take part in the encounter?

■ How did it change your relationship with your patient or colleagues?

■ How did you feel at the end?

● Mindful Breaths x3

Session # 4

● Mindful Breaths x3

● Checking in with each other.

○ How are you feeling at this moment?

○ Are you “awake’?

○ Are you distracted? Why?

● Understanding Empathy and Compassion

○ Definitions of Empathy and Compassion

○ What are the differences?

○ How are they related?

○ How does each differently address suffering?

○ How can we reframe our approach from being empathic to more compassionate? ○ Do you have compassion for yourself? When was the last time you were kind to yourself?

○ Think about the last time you were judgmental with yourself? What did that feel like?

○ Think about a time when you were non-judgmental with yourself? What did that feel like?

● Mindful Breaths x3

Session # 5

● Mindful Breaths x3

● Checking in with each other.

○ How are you feeling at this moment?

○ Are you “awake’?

○ Are you distracted? Why?

- Finding meaning in the everyday

○ What brought you to the work that you’re doing now, to your profession? ○ Do you find satisfaction at work? Why or Why not?

○ How does your work speak to your passions and how does it use the gifts that you have to offer?

○ How do you use your passions and gifts to bridge your personal and professional spaces?

○ **Work is our conversation with the world,** where we are invited to engage and interact with our colleagues, patients and strangers. How does that resonate with you?

○ It’s not just the importance of the work we produce but also what the work produces for us. How does being in relationship with our colleagues, patients and strangers support this? Good work comes from people working together and encouraging one another, finding a place of connection.

● Mindful Breaths x3
